# A neural network for the detection of soccer headers from wearable sensor data

**DOI:** 10.1038/s41598-022-22996-2

**Published:** 2022-10-28

**Authors:** Jan Kern, Thomas Lober, Joachim Hermsdörfer, Satoshi Endo

**Affiliations:** 1 Department of Sport and Health Sciences, Chair of Human Movement Science, Technical University of Munich, Munich, Germany; 2 TUM School of Computation, Information and Technology, Chair of Information-Oriented Control, Technical University of Munich, Munich, Germany

**Keywords:** Trauma, Risk factors, Machine learning

## Abstract

To investigate the proposed association between soccer heading and deleterious brain changes, an accurate quantification of heading exposure is crucial. While wearable sensors constitute a popular means for this task, available systems typically overestimate the number of headers by poorly discriminating true impacts from spurious recordings. This study investigated the utility of a neural network for automatically detecting soccer headers from kinematic time series data obtained by wearable sensors. During 26 matches, 27 female soccer players wore head impacts sensors to register on-field impact events (> 8 g), which were labelled as valid headers (VH) or non-headers (NH) upon video review. Of these ground truth data, subsets of 49% and 21% each were used to train and validate a Long Short-Term Memory (LSTM) neural network in order to classify sensor recordings as either VH or NH based on their characteristic linear acceleration features. When tested on a balanced dataset comprising 271 VHs and NHs (which corresponds to 30% and 1.4% of ground truth VHs and NHs, respectively), the network showed very good overall classification performance by reaching scores of more than 90% across all metrics. When testing was performed on an unbalanced dataset comprising 271 VHs and 5743 NHs (i.e., 30% of ground truth VHs and NHs, respectively), as typically obtained in real-life settings, the model still achieved over 90% sensitivity and specificity, but only 42% precision, which would result in an overestimation of soccer players’ true heading exposure. Although classification performance suffered from the considerable class imbalance between actual headers and non-headers, this study demonstrates the general ability of a data-driven deep learning network to automatically classify soccer headers based on their linear acceleration profiles.

## Introduction

With more than 265 million active participants across the globe, the game of soccer is the most popular sport worldwide^1^. While playing soccer is associated with both physical and psychosocial health benefits, as a contact sport, it also poses players at an increased risk for sustaining impacts to the head. In recent years, there has been growing concern about the potential long-term consequences of these sport-related head impacts, especially in light of the proposed association between recurrent concussions and the late development of neurodegenerative disease, such as dementia or chronic traumatic encephalopathy (CTE)^2,3^. When compared to other types of contact sports like American football or rugby, concussion rates in soccer are relatively low^4^. As a unique feature of the game, however, soccer players actively expose themselves to repetitive head impacts (RHI) by purposefully heading the ball in order to control, deflect, and redirect it in play. While impacts resulting from the execution of a single header, typically, are of subconcussive nature and, therefore, do “not result in a known or diagnosed concussion on clinical grounds”^5^, a debate on the potentially adverse cumulative effects associated with soccer heading has emerged within the scientific and public community. Within this context, it is hypothesized that repetitive heading, over several years of play, might lead to cognitive, emotional, and behavioral deficits or even contribute to the development of CTE^6,7^. However, even though a dose–response relationship between cumulative heading over a single season and the degree of cognitive dysfunction has been suggested^8,9^, evidence of long-term consequences due to repetitive soccer heading remains inconclusive as the entire history of head impacts is typically not considered within these studies^10–12^.

To investigate the potentially adverse effects of RHI due to soccer heading, a reliable quantification of long-term individual heading exposure is crucial. Next to video-based observation of play, which is considered the gold standard^13,14^, recent technological advances have contributed to the development of wearable sensor technologies that enable the study of head impacts, along with their associated kinematics, in vivo. As these sensors allow for an automatized registration and characterization of impact events, they have proven useful for studying long-term exposure to RHI and, thus, constitute a popular means for the assessment of soccer players’ individual heading frequency^15–17^. However, various studies have shown that available sensor systems suffer from poor accuracy in differentiating true head impacts from other acceleration events, such as jumping or manual contacts with the sensor, that exceed the sensors’ pre-defined trigger threshold (typically 10 g). Consequently, recorded impact data are prone to contain a large number of false positive events^18–20^. In an attempt to minimize the number of false positive recordings, some authors suggested to increase the sensors’ pre-set trigger to 16 g^19^ or even 20 g^21^ in order to selectively capture meaningful head impacts. However, since many soccer headers result in head accelerations well below these thresholds^18,22^, a considerable proportion would be missed by the sensor when employing these recommendations^15,17^. Moreover, manufacturers equipped their sensors with proprietary processing algorithms to detect true impacts and remove “spurious” events^19^. While little is known about their exact working principle, the results of several studies indicate that these algorithms, still, not only face problems with rejecting false positives, but also struggle to accurately identify and include true head impacts, thus leading to false negative events^21,23,24^. Accordingly, head impact events recorded by wearable sensors have to be verified through comparison with other independent sources of information, such as video analysis^16^. While video confirmation of sensor data constitutes a reliable means to verify on-field recordings, this task is time consuming and tedious and, thus, prospective approaches aiming to investigate the potential association between RHIs and adverse health outcomes are typically restricted to the examination of rather small cohorts over a limited period of time.

Since the kinematic waveforms of true head impacts, e.g., soccer headers, and spurious acceleration events typically look distinct, within recent years, a modest number of studies has focused on the development of data-driven machine learning models to automatically classify sensor-recorded head impact events based on their characteristic acceleration or velocity profiles. In a non-purely sporting context, Rooks et al.^25^ trained a simple decision tree to distinguish head impacts from other acceleration events during routine sparring sessions of U.S. Army combat soldiers. While their algorithm was able to correctly classify 88% of all sensor-recorded events, only half (51%) of the predicted impacts actually corresponded to actual head impacts (precision)^25^. In actual sporting contexts, most approaches focused on the discrimination between true head impacts and non-impacts in American^26,27^ or Australian rules football^28^. While the models employed by Wu et al.^26^ (support vector machine, SVM) and Gabler et al.^27^ (Adaboost) achieved classification performances ranging from 68.5% to 93.8% and 81.6% to 98.3%, respectively, the best-performing classifier (XGBoost) in the study of Goodin et al.^28^ showed promising impact and non-impact detection rates of approx. 95%. Besides the above-described works, two studies also used machine learning classifiers for distinguishing between true impact events and non-impacts from kinematic sensor data in the game of soccer. On a dataset of youth soccer athletes, the learning system of Motiwale et al.^29^ correctly detected 88% of actual impact events (sensitivity), whereas only 47% of non-impacts were identified as such (specificity). More recently, DiCesare et al.^30^ reported an improved classification performance of 84% sensitivity and 83% specificity when testing their classifier on a dataset of high school soccer players. Although these works further demonstrate the potential of intelligent, data-driven techniques for the detection of soccer-related impacts from wearable sensor data, none of them explicitly focused on the detection of header events but also considered other types of impacts, including body collisions or ground contacts, triggering the sensor. As the kinematic waveforms of these contacts might substantially differ from those of direct head impacts from heading the ball, their inclusion may have affected classification performance.

While in the mentioned studies, the majority of classifiers relied on feature-based approaches that require an a-priori selection of appropriate features, neural networks that automatically extract relevant features from kinematic time series data might constitute a promising tool for detecting and classifying head impacts. Especially deep learning approaches, such as Long Short-Term Memory (LSTM) networks, appear to be specifically suited for time series classification, as previously demonstrated in the context of human activity recognition from wearable sensor data^31,32^. Based on this, we assumed that an LSTM neural network should be able to precisely differentiate between sensor-recorded headers and non-header events. By building on the need of a reliable quantification of soccer heading exposure, this study investigated the utility of an LSTM neural network for an automatized identification and classification of soccer heading events from wearable sensor data. To this aim, we used a wearable head impact sensor to record three-dimensional linear acceleration at the head experienced by players from a semi-professional female soccer team across nearly two seasons. Ground truth header and non-header datasets were obtained from synchronized video recordings in order to train, validate, and test an LSTM neural network. With this data-driven method, we expected to achieve a more accurate detection of soccer headers from wearable sensor data compared to previous approaches.

## Methods

Head impact data was collected from 27 players (mean age: 21.8 ± 4.1 years) of a semi-professional female soccer team (German 3rd division) during 26 competitive matches throughout the 2017/18 and 2018/19 seasons. All subjects provided written informed consent to participate in the study, which was approved by the ethical committee of the School of Medicine of the Technical University of Munich and conducted in accordance with the declaration of Helsinki.

### Data acquisition

For the collection of header data, individually assigned head impact sensors (xPatch, X2 Biosystems, Seattle, USA) were deployed to all athletes involved. The xPatch device (dimensions: 37 mm x 14 mm) is the most widely used head impact sensor for application in un-helmeted sports^23^ and has been independently validated in previous studies^33,34^. Before warming up to each match, wearable sensors were switched on and consistently affixed behind the players’ right ear on the skin covering the mastoid process by means of a double-sided adhesive cloth tape. The xPatch sensors comprise a triaxial accelerometer, sampling at a frequency of 1000 Hz, to record linear acceleration of the head in anterior–posterior (x), left–right (y), and inferior-superior (z) direction. If, during matches, an event exceeded a predefined accelerometer reading of 8 g, data acquisition was triggered and linear acceleration time series data were recorded for 100 ms (10 ms pre-trigger, 90 ms post-trigger) and stored internally. By expecting soccer headers to result in impact magnitudes even below the commonly applied 10 g trigger threshold, we opted for this comparatively lower threshold in order to capture as many header events as possible. Immediately after each match, sensor data were downloaded on a PC by proprietary software (Impact Monitoring System, X2 Biosystems) and stored for analysis. The software also classified each sensor-recorded event using an internal algorithm. While not being publicly available, this algorithm analyzes several factors of the impacts’ acceleration waveforms, such as area under the acceleration curve and the number of data points above the predefined threshold^21^ in order to categorize any given impact event as either *Hit* (true head impact) or *Clack* (spurious impact). To be able to independently verify sensor-recorded impact data, video footage of all matches was captured by means of two high-definition video cameras (Sony HDR-CX653, Sony Corp., Tokyo, Japan) recording 50 fps at 1080p resolution. Prior to the matches, each camera was mounted on a tripod which was, depending on local conditions, located on opposing sides of the pitch near the midpoint with each of them capturing one half of the pitch. Immediately before the start and after the end of each half, a world clock was displayed to obtain the exact start and end times of each match. Simultaneously, a “dummy” sensor was struck five times in full view of the camera to allow for an exact temporal synchronization of video footage and sensor data.

### Data processing

To obtain ground truth labels for the collected sensor data, video analysis was used to cross-verify sensor-recorded header events. First, video analysis of each match was carried out by a trained researcher using Kinovea 0.8.27 (https://www.kinovea.org) to identify header events. By instructing the reviewer to have high sensitivity in recognizing any instant in which players’ heads had direct contact to the ball, each video-observed header, along with its associated time stamp, was assigned to the respective player. If a header could only be suspected but not unequivocally identified on video, it was not considered for further steps of data processing. In addition to the mere detection of header events, the researcher was also obliged to register unintentional head impacts that might have occurred during a header, such as head-to-head or elbow-to-head contacts. However, no such events could be identified during the review process. To ensure a single rater was appropriate for the analysis of video data, a subset of five matches was randomly selected to be reviewed and analyzed by a second independent researcher. The interrater reliability in identifying players’ individual numbers of headers was assessed by computing the intraclass correlation coefficient (ICC).

Next, sensor recordings were precisely matched to the video-observed headers based on the sensor data’s date and event time stamp. As proposed by Kuo et al.^16^, we used a time window of ± 2 s to select header events from the sensor data, i.e., any sensor recording that occurred within 2 s of a video-identified header was assigned to the respective event. According to this, a valid header (VH) was defined as any video-identified header for which a corresponding sensor event could be determined. Extraction of the ground truth non-header dataset was conducted as follows. First, sensor-recorded events outside of verified match times, as identified by the exact start and end times of each match, were excluded. Then, video footage of each match was reviewed to determine exact time points at which specific players were substituted in order to compile a list of players on the pitch by time of each match. All sensor events that were associated with players outside the pitch were removed. Consequently, a ground truth non-header (NH) was considered as any sensor-recorded event that was associated with players on the pitch during verified match times but did not correspond to a video-observed header.

### Implementation of the neural network

**Figure 1:**
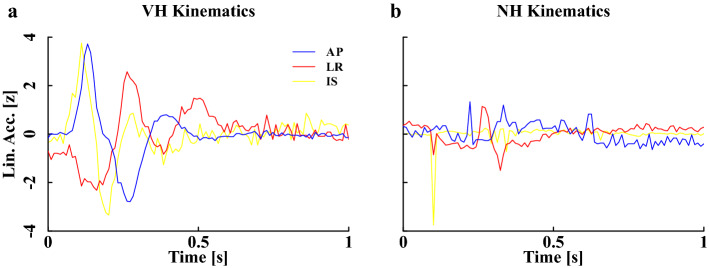
Normalized kinematics of an example VH (**a**) and NH (**b**). AP: anterior-posterior; LR: left-right; IS: inferior-superior.

**Figure 2:**
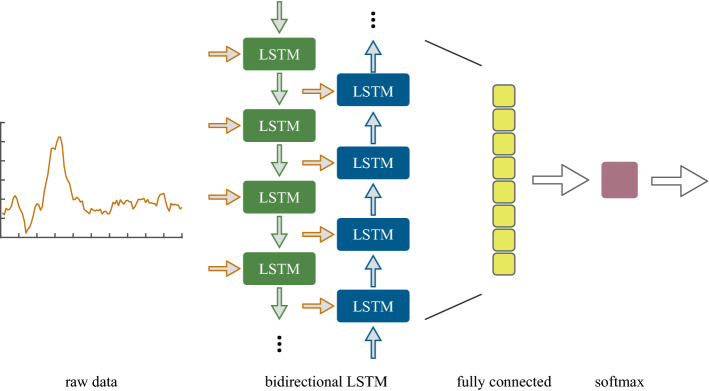
Architecture of the proposed LSTM neural network.

To differentiate VHs from NH events, we trained a Long Short-Term Memory (LSTM) neural network using MATLAB R2019a (The MathWorks, Natick, USA). As a subset of recurrent neural networks, LSTM networks are specifically designed to learn dependencies between individual time steps of sequence data in order to make predictions based on the sequences’ characteristic patterns. Z-normalized raw linear acceleration profiles of VHs and NHs (Fig. 1) were used as input sequences to the LSTM network. Since acceleration magnitudes varied substantially within the two classes, normalization of the input data ensures that the classification is rather based on structural similarities/dissimilarities than on amplitude-driven ones. The core components of the here proposed neural network are the sequence input layer and the bidirectional Long Short-Term Memory (BiLSTM) layer, which are the first two layers of the network. While the input layer inserts the three-dimensional time series data (sequence length: 100) into the network, long-term dependencies between single steps of the full time series are learned within the BiLSTM layer, which consists of 100 LSTM cells and outputs the last element of a sequence. The number of hidden units corresponds to the amount of information remembered between time steps (the hidden state). The outputs of the two core layers were flattened and then fed into a fully connected layer, in which all neurons are connected to the ones of the previous BiLSTM layer. Within the fully connected layer, the two target classes (VH and NH) are specified. Specifically, the input is multiplied by a weight matrix ${W}$ and a bias vector ${b}$ is added. The fully connected layer acts independently at each time step. Thus, if the BiLSTM layer outputs an array ${X}$ of size ${D}$-by-${N}$, where ${D}$ denotes the three axes of accelerometer data (x, y, z) and $N$ represents the length of the acceleration sequence (i.e., 100), the fully connected layer outputs an array ${{Z}}$ of size $2$-by-${{N}}$. At time step ${{t}}$, the corresponding entry of ${{Z}}$ is ${{W}}{{{X}}}_{{{t}}}+{{b}}$, where ${{{X}}}_{{{t}}}$ denotes time step ${{t}}$ of ${{X}}$. Subsequently, a softmax layer applies the normalized exponential (softmax) function to the output of the fully connected layer to obtain the probability distribution over the target classes. Finally, the binary cross entropy loss function was used for classification. The above-described network architecture is depicted in Fig. 2.

### Training and optimization

For training and validation of the neural network, randomly selected partitions of 49% and 21% of the entire ground truth dataset were used, respectively. Since training the network on an unbalanced dataset (i.e., unequal number of VHs and NHs) would bias the classification output towards a more sensitive detection of the majority class, we used augmentation to increase the number of VHs in the training and validation set to obtain a class ratio of one. Augmentation was performed by adding pink noise multiplied with ${{\sigma}}$ = 10% to randomly selected VH time series of the respective dataset.

Adaptive moment estimation (Adam) was used as an optimizer for training the LSTM network. As an extension of stochastic gradient descent, Adam computes adaptive learning rates for each of the network’s parameters (weights and biases) by using estimates of first and second moments of the gradients^35^. Specifically, the algorithm calculates an exponentially decaying moving average of both the parameter gradient and the squared gradient,

1 $${{{m}}}_{{{l}}}={{{\beta}}}_{1}{{{m}}}_{{{l}}-1}+(1-{{{\beta}}}_{1})\nabla \mathbf{\rm E}({{{\theta}}}_{{{l}}})$$

2 $${{{v}}}_{{{l}}}={{{\beta}}}_{2}{{{v}}}_{{{l}}-1}+\left(1-{{{\beta}}}_{2}\right){\left[\nabla \mathbf{\rm E}\left({{{\theta}}}_{{{l}}}\right)\right]}^{2}$$

where $\mathbf{\rm E}({{\theta}})$ is the loss function to be minimized, ${{\theta}}$ is the parameter vector, ${{l}}$ is the iteration number, and ${{{\beta}}}_{1}$ and ${{{\beta}}}_{2}$ represent the decay rates of the moving averages, which were set to 0.9 and 0.99, respectively. These moving averages are used to update the network parameters as

3 $${{{\theta}}}_{{{l}}+1}={{{\theta}}}_{{{l}}}-\frac{{\alpha{{m}}}_{{{l}}}}{\sqrt{{{{v}}}_{{{l}}}}+{{\epsilon}}}$$

where $\alpha$ is the learning rate and ${{\varepsilon}}$ represents a smoothing term, which was set to 10^–8^ to avoid division by zero. By using a moving average, information about previous gradients is retained, thus enabling the parameter updates to pick up momentum as gradients remain similar over multiple iterations. In turn, if the gradients mostly contain noise, the moving average, and so the parameter updates, become smaller. The number of epochs for training the network was set to three, as determined by comparing training and validation sensitivity over 20 epochs.

Network hyperparameters comprised the initial learning rate (testing range: 1e^−2^–1), mini-batch size (testing range: 64–128), the factor for L2-regularization (testing range: 1e^−8^–1e^−1^) as well as the number of nodes within the fully connected layer (testing range: 20–100). Optimization of these parameters was performed using Bayesian optimization. To find the hyperparameter settings that minimize the objective function ${{f}}({{x}})$, i.e. the validation error, Bayesian optimization uses prior information about ${{f}}$ to approximate the objective function with a probabilistic Gaussian Process model^36^. As this surrogate model is much cheaper to evaluate than the true objective function, hyperparameter configurations that are likely to yield an improvement over previous results are determined by numerically optimizing the criterion of Expected Improvement (EI) of the surrogate model and are subsequently used to evaluate the actual objective function^37,38^. After each evaluation, the observed result is, again, used to update the surrogate model in order to better approximate the true objective function and to determine the next set of hyperparameters to evaluate. In summary, for a set of hyperparameters ${{{x}}}_{{{t}}}$, the objective function is evaluated at ${{{x}}}_{{{t}}}={{{a}}{{r}}{{g}}{{m}}{{a}}{{x}}}_{{{x}}}{{u}}({{x}}|{{{D}}}_{1:{{t}}-1})$, where ${{u}}$ represents the EI of the surrogate and ${{{D}}}_{1:{{t}}-1}=({{{x}}}_{{{t}}-1},{{{y}}}_{{{t}}-1})$ denote the ${{t}}-1$ samples drawn from ${{f}}$ so far. To limit optimization time, the maximum was set to five hours. The hyperparameter configuration yielding the lowest classification error on the validation set was used to classify the test samples.

### Evaluation

To allow for an unbiased evaluation of the learning system, testing was performed on the remaining 30% of the entire dataset that have not been used for training or validation. Unlike Wu et al.^26^, who used sensor data from a pre-defined subset of American football matches for independently testing their classifier, we opted for randomly allocating sensor events from all matches to our particular datasets. Evaluation was based on the total number of true positives (TP), false positives (FP), true negatives (TN), and false negatives (FN). Within this context, TP represents a header that was correctly classified as such, FP is a non-header that was incorrectly classified as a header, TN is a non-header that was correctly classified as a non-header, and FN denotes a header that was incorrectly classified as a non-header. These numbers were used to evaluate the classification performance by calculating sensitivity, specificity, precision, accuracy, and the F1-score, which are defined as follows:

4 $$Sensitivity=\frac{TP}{TP+ FN}$$

5 $$Specificity=\frac{TN}{TN+FP}$$

6 $$Precision=\frac{TP}{TP+FP}$$

7 $$Accuracy=\frac{TP+TN}{TP+FP+TN+FN}$$

8 $$F1=2*\frac{Sensitivity*Precision}{Sensitivity+Precision}$$

Of these metrics, sensitivity and precision were considered the most important indicators of the learning system’s classification performance. Sensitivity resembles the network’s ability to identify true header events while precision indicates the proportion of these events that is correctly classified. Consequently, the neural network’s sensitivity and precision can be used to determine athletes’ heading exposure.

In an initial step, we assessed the LSTM network’s general ability to distinguish between true headers and non-header events. For this purpose, we used a balanced test dataset comprising an equal number of VHs and NHs. In a second scenario, we aimed to evaluate the system’s classification performance on highly unbalanced datasets, as typically obtained in real-life settings^20,21^. The VH/NH ratio of approx. 1/21 that we observed in the present study (see below) was used for this second test set. To obtain an unbiased estimate of the network’s classification performance, fivefold cross validation^39^ was used for both scenarios and the average taken as the final result.

To further compare our learning system to the commonly employed approach of increasing the sensor’s linear acceleration threshold as well as to the sensor’s proprietary filtering algorithm, we computed the above-described performance metrics for the following classification methods.

1. LSTM. As described above.
2. Linear acceleration thresholding (10 g). In this classification method, all sensor-recorded events with peak linear acceleration magnitude greater than 10 g are classified as VHs. In previous research, a threshold of 10 g has been most commonly applied to study soccer heading frequency^18,23^.
3. Linear acceleration thresholding (16 g). Similar to the previously described approach, all sensor events with peak linear acceleration magnitude greater than 16 g are classified as VHs. Increasing the pre-set trigger threshold to 16 g (or higher) has been suggested to only capture meaningful head impacts^19,21^.
4. The sensor’s proprietary classification algorithm (X2). Here, only sensor-recorded events that have been labeled as Hit by the sensor’s internal algorithm are considered VHs.

## Results

ICC analysis (κ = 0.95, 95% CI [0.92–0.96]) revealed an excellent^40^ interrater agreement for the identification of header events from video data. Consequently, we considered a single rater to be appropriate for this task.

**Figure 3:**
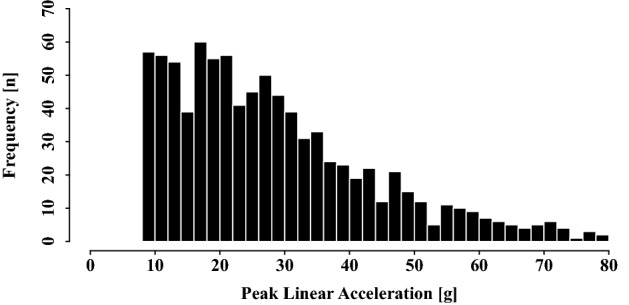
Frequency of headers across different impact magnitudes.

Analysis of video footage yielded a total of 1016 header events that involved an instrumented player wearing a sensor. In contrast, 44,481 impacts were recorded by the xPatch sensors at the pre-selected 8 g trigger threshold throughout the 26 match days. Removing sensor events outside of verified match times and impacts associated with players not on the pitch resulted in a dataset of 20,049 sensor recordings, of which 904 (i.e., 89.0% of header events determined on video) could be unequivocally assigned to video-identified headers. Consequently, these 904 sensor events were considered VHs, whereas the remaining 19,145 recordings were considered ground truth NHs. The frequency distribution of VHs across different magnitudes is depicted in Fig. 3.

Tuning of the network’s hyperparameters by means of Bayesian optimization revealed that the lowest classification error was to be achieved with an initial learning rate of 0.168, a mini batch size of 119, an L2 regularization factor of 0.0016, and 43 nodes within the network’s fully connected layer. Therefore, this configuration was retained in order to independently classify the test samples.

**Figure 4:**
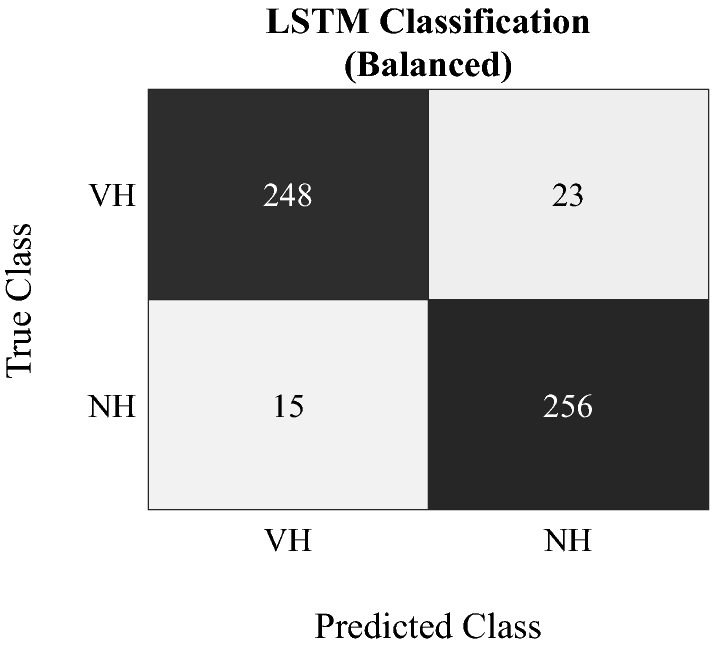
Confusion matrix for the classification of sensor data into soccer headers (VH) and non-headers (NH) with the proposed LSTM neural network.

The general ability of the developed LSTM neural network to differentiate between actual headers and non-header events was assessed using a balanced test dataset comprising 30% of the total number of headers, i.e., 271 VHs and a randomly selected equal-sized subset of 271 NHs. The network’s average classification performance and the corresponding confusion matrix are displayed in Table 1 and Fig. 4, respectively. By reaching values of more than 90% across all metrics, the deep learning model demonstrated very good overall classification performance. In detail, the learning system achieved 91.5% sensitivity, 94.5% specificity, 94.3% precision, 93.0% accuracy, and an F1-score of 92.9%.

**Table 1:** Evaluation of the LSTM neural network’s classification performance on a balanced dataset (271 VHs, 271 NHs).

	LSTM (balanced) (%)
Sensitivity	91.5
Specificity	94.5
Precision	94.3
Accuracy	93.0
F1	92.9

To evaluate the learning system’s ability to detect and classify header events in unbalanced datasets, as typically encountered in real-life settings, testing was further performed on the whole set of 271 VHs and 5743 NHs. The average classification performance of the LSTM neural network as well as the respective performances that were achieved by employing the other approaches (10 g, 16 g, X2) are depicted in Table 2.

**Table 2:** Evaluation of the different header classification approaches.

	10 g threshold	16 g threshold	X2	LSTM
Sensitivity	**93.7** %	77.3%	78.8%	90.3%
Specificity	56.6%	86.0%	87.0%	**94.2**%
Precision	9.3%	20.7%	22.2%	**42.2**%
Accuracy	58.3%	85.6%	86.6%	**94.0**%
F1	16.8%	32.6%	34.6%	**57.5**%

Values printed in bold represent the highest score across approaches.

**Figure 5:**
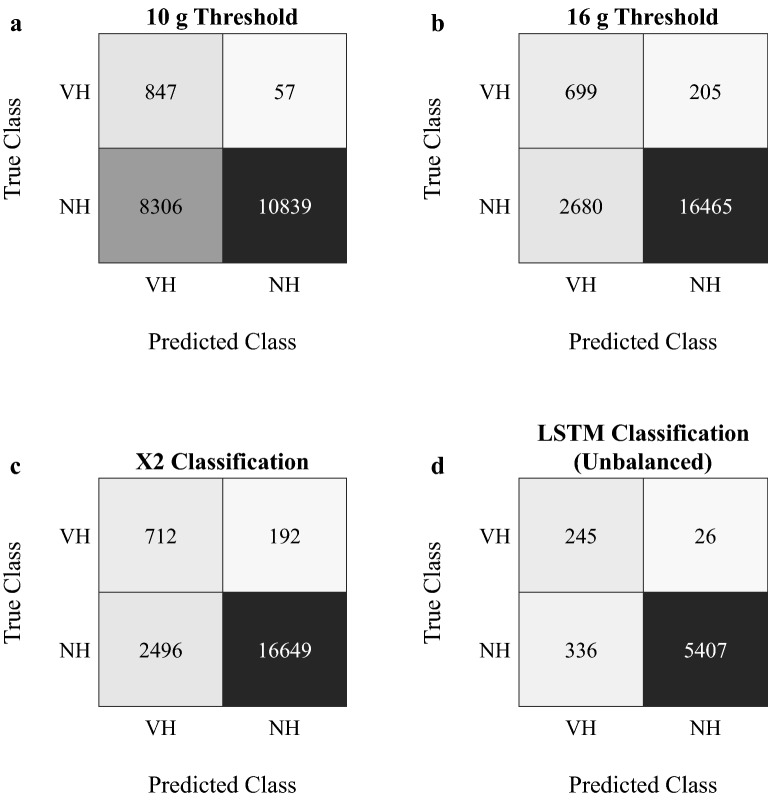
Confusion matrices for the classification of sensor data with 10 g acceleration threshold (**a**), 16 g threshold (**b**), X2’s proprietary algorithm (**c**), and the here proposed LSTM learning system (**d**).

Without any further processing (8 g threshold only), the head impact sensor achieved a precision score of 4.5%, meaning that 95.5% of all sensor-recorded impacts did not correspond to ground truth header events. While precision slightly increased from 9% (10 g) to 20% (16 g) and 22% (X2) when employing the threshold-based methods and the sensor’s proprietary algorithm, the highest sensitivity score (93.7%) across all approaches was achieved using the 10 g linear acceleration threshold. However, this high header detection rate was accompanied by the lowest specificity value of only 56.6%. In summary, although classification tended to be more balanced when moving from 10 to 16 g to X2, our proposed LSTM neural network clearly outperformed the previous approaches by reaching classification scores of 90.3% sensitivity, 94.2% specificity, 42.2% precision, 94.0% accuracy, and an F1-score of 57.5%. The confusion matrices for the different classification approaches are displayed in Fig. 5.

## Discussion

To allow for an accurate investigation of the potentially detrimental effects of purposeful soccer heading on brain health, a reliable quantification of athletes’ heading exposure is crucial. In this paper, we present a data-driven method for an automatized detection and classification of soccer headers based on their kinematics as obtained by wearable head impact sensors. By cross-verifying on-field sensor recordings with independent video analysis, we obtained ground truth training and validation data for a Long Short-Term Memory (LSTM) neural network, which was then used to classify impact events into valid headers (VH) and non-headers (NH) based on their characteristic 3D linear acceleration profiles. When tested on a balanced dataset, our learning system demonstrated very good overall classification performance by achieving scores of more than 90% across all calculated metrics. And although we observed a drop in system performance, especially with respect to classification precision, when testing was performed on an unbalanced dataset, our learning system still demonstrated superior performance as compared to the sensor system’s proprietary algorithm as well as to the commonly applied technique of using an increased linear acceleration threshold for distinguishing between VHs and NHs.

Next to video-based or direct observation of play and self-report techniques, wearable head impact sensors constitute a popular means for the assessment of soccer players’ individual heading exposure^15–17^. In the present study, the xPatch sensors captured 904 of 1016 video-identified header events (89% sensitivity) at a pre-determined linear acceleration threshold of 8 g, indicating their potential for an automatized detection of soccer headers in on-field scenarios. However, in line with previous reports^19,21^, comparison of video and sensor data revealed a large number of false positive sensor recordings resulting in precision and accuracy scores of only 4.5%, respectively. Thus, simply relying on these sensor data would lead to a substantial overestimation of soccer players’ actual heading exposure and ultimately introduce bias to any investigation of a potential dose–response relationship between soccer heading and adverse health outcomes^17^. This, once again, illustrates the need to additionally verify or filter sensor recordings by secondary sources of information in order to obtain reliable exposure data^16,17^. Since previous studies demonstrated the potential of data-driven machine learning models for the identification of various types of head impacts in both American^26,27^ and Australian rules football^28^ as well as soccer^29,30^, we used an LSTM neural network for the automatized detection of header events as the most common type of RHI in the game of soccer^19,23^.

When applied to classify sensor-recorded events in a balanced dataset of 271 VHs and NHs, our learning system demonstrated a very high overall classification performance (> 90% across all metrics). More specifically, 248 of 271 headers were correctly identified as such (91.5% sensitivity) while only 15 of 263 events were erroneously classified as headers (94.3% precision). These results provide evidence for our neural network’s general ability to not only identify true headers, but also to detect and remove spurious sensor recordings based on their characteristic 3D linear acceleration profiles. When compared to previous studies, only Wu et al.^26^ and Gabler et al.^27^ tested their models on (almost) balanced datasets, but, within this context, focused on the detection of head impacts in American football. At impact/non-impact ratios of 1/1 and approx. 1/1.3, the authors reported similarly good classification performances of up to 93%^26^ and 98%^27^, respectively. While these works relied on feature-based approaches, which require an a-priori selection of appropriate features from time series, we successfully used a deep LSTM neural network, which automatically generates and extracts relevant features, including temporal information underlying the recorded kinematic time series data, to detect and classify header events in the game of soccer.

Consistent with previous findings^20,21^, we observed that, even after removing sensor recordings outside verified match times and events associated with players not on the pitch, the vast majority (> 95%) of registered sensor events did not result from purposeful heading of the ball. Therefore, we examined whether our neural network could still produce an accurate estimate of heading exposure in case of highly unbalanced data by further testing the learning system on a dataset with the here observed VH/NH ratio of approx. 1/21 and comparing its classification performance to commonly applied threshold-based methods as well as to the sensor’s proprietary algorithm. By achieving more than 90% sensitivity, our LSTM network confirmed its ability to reliably detect true header events. Accordingly, less than one out of ten VHs was incorrectly classified as NH. While the simple use of a 10 g linear acceleration threshold provided an even higher sensitivity score of 93%, this came to the expense of erroneously identifying 43% of NHs as true headers. The xPatch sensor’s proprietary classification algorithm only achieved 79% sensitivity, which corresponds to a false negative rate of over 20%. This is in line with previous research, in which similar false negative rates have been reported for different types of currently available head impact sensors in both laboratory^24^ and on-field settings^23^.

Concerning the identification and rejection of NHs from an unbalanced dataset, the developed LSTM classifier achieved a specificity score of more than 94% and, thus, outperformed the commonly applied threshold-based methods (10 g: 56%, 16 g: 86%) as well as the sensor’s proprietary classification algorithm (87%). While this illustrates the network’s general ability to correctly remove false positive sensor recordings, precision should be preferred over specificity to assess the potential of a learning system to exactly quantify heading exposure. This is due to the great amount of NHs (i.e., true negatives) in unbalanced field data, which can result in high specificity even with a substantial amount of false positives^26,41^. Despite only 336 (< 6%) out of 5743 NHs were incorrectly classified as VHs and even though our proposed LSTM neural network performed better than the other approaches by achieving a precision score of 42% (10 g: 9%; 16 g: 21%; X2: 22%), players’ true heading exposure would have been overestimated by a considerable margin. The two studies^29,30^ that focused on the detection of soccer-related head impacts from wearable sensor data also used unbalanced datasets to evaluate their models’ classification performance. When compared to our results, the classifiers of Motiwale et al.^29^ (impact/non-impact ratio: 1/5) and DiCesare et al.^30^ (impact/non-impact ratio: 1/4) achieved slightly lower sensitivity scores of 88% and 83%, while reaching precision values of 26% and 57%, respectively. As the comparatively higher precision reported by DiCesare et al^30^ may be attributable to the more favorable distribution of true impacts and non-impacts, it can be argued that, although still struggling to successfully remove false positive recordings, the here proposed LSTM neural network outperformed previous approaches that aimed to detect head impacts from wearable sensor data in the game of soccer. A potential explanation for the superior performance of our learning system might lie in the fact that both Motiwale et al.^29^ and DiCesare et al.^30^ considered other types of impacts, such as ground contacts or body collisions, triggering the sensor and that the kinematic waveforms of these events might substantially differ from the characteristic acceleration profiles of direct head impacts from heading the ball. Taken together, our findings are in line with previous studies by further demonstrating the ability of intelligent, data-driven approaches in differentiating between true headers and non-header events – especially when compared to the employment of sensor manufacturers’ proprietary classification algorithms^15^. However, to exploit the general potential of these models for an automatized detection and classification of soccer headers, future research should focus on the development of learning systems specifically able to handle the considerable class imbalance, which is typically to be observed in sensor data collected in on-field scenarios.

While this imbalance of true headers and spurious events in field-recorded sensor data has been commonly reported^20,21^, previous research suggested to increase the sensor’s pre-set trigger threshold to 16 g^19^ or even 20 g^21^ in order to reduce false positive recordings. Although our results, in line with the findings of Press and Rowson^18^, indicate that this approach can lead to a more balanced distribution of VHs and NHs and an increase in classification precision, soccer headers have been shown to frequently result in head accelerations below 16 g, which would go unrecognized when selecting these increased thresholds^15,17,18,22^. This notion is confirmed by the present results as almost 25% of VHs were missed when employing an increased acceleration threshold of 16 g compared to our pre-defined 8 g trigger. Moreover, a substantial number of headers (57/904 = 6.3%) came along with even lower sensor readings between 8 and 10 g (Fig. 3). Next to confirming our rationale of choosing a rather low acceleration threshold in order to capture as many header events as possible, these findings, again, demonstrate the inadequacy of using a simple linear acceleration threshold for distinguishing between VHs and NHs. In turn, the results reemphasize the necessity to verify sensor-recorded head impact data by a secondary source of information^16,17^. While video review constitutes the current gold standard for the confirmation of sensor data, in the future, an automatized machine learning classifier may constitute a promising alternative to time-consuming and resource-intensive analyses of video footage. And although Rezaei and Wu^42^ just recently introduced a novel computer vision algorithm for an automatized detection of soccer headers from match videos, learning systems, like the here proposed LSTM neural network, may still improve head impact verification by addressing some of the common limitations associated with both manual and automatized video analysis, such as poor video quality or periods of obstructed view, in which potential header events might not be unequivocally identified^15^. In addition, sensors also measure impact magnitude, which can only be inaccurately derived from video recordings. In this sense, our developed LSTM neural network can serve as a valuable step towards a post-processing tool for an automatized and reliable detection and classification of soccer headers based on their characteristic linear acceleration profiles.

Although constituting a promising tool for enabling an automatized quantification of soccer headers in on-field scenarios, in its current form, the here proposed LSTM classifier is only evaluated to be used for the detection and classification of soccer headers with the specific sensors used in this study. For other devices with electrical or mechanical properties different to those of the xPatch, adaptation and retraining might be required. Moreover, training of the learning system was performed using data from female soccer players. In order to improve and validate the classifier’s independent performance and, in this sense, refine it for future field use, additional training should be carried out with an increased dataset comprising header data from both male and female as well as youth soccer players.

In contrast to previous studies^26,27,41^, in which identification of head impacts was carried out based on linear acceleration and rotational velocity features, our proposed LSTM neural network for the detection of soccer headers solely relied on automatically extracted features from linear acceleration time series data. However, as Wu et al.^26,41^ as well as Gabler et al.^27^ identified rotational velocity features that appeared to distinguish between actual head impacts and non-impacts, we performed an additional analysis in which we trained our learning system with both linear acceleration and rotational velocity profiles of VHs and NHs in order to determine whether this inclusion of new information might potentially increase the network’s classification performance. While we observed a subtle increase in sensitivity, all other metrics slightly decreased when impact classification was based on both linear acceleration and rotational velocity features (Supplementary Fig. S1 and Supplementary Table S1). Overall, however, changes in classification performance were negligible when adding rotational velocity features. There might be several explanations for this observation: firstly, the mentioned studies^26,27,41^ that opted for the inclusion of rotational velocity or acceleration features for automatically differentiating between actual head impacts and non-impacts were conducted in the context of helmeted sports. Since we did not observe an increase in classification performance when including gyroscope data to our analysis, it might be argued that head impact characteristics from soccer headers might differ from those of helmet impacts, as typically observed in American football, and that header-related impacts may be described by linear acceleration rather than rotational velocity features. Secondly, in contrast to our approach, all Gabler et al.^27^ and Wu et al.^26,41^ used an instrumented mouthguard to capture head impact kinematics. Therefore, these differences in device form factor and sensor location might provide another explanation for the almost unchanged classification results when adapting our LSTM neural network to also use rotational velocity features for distinguishing between VH and NH events. Within this context, it also has to be noted that especially the rotational velocity data measured by the xPatch have been shown to be highly imprecise and erroneous^33,34^, which might be attributable to the comparatively low gyroscope sample rate of 850 Hz. Consequently, the obtained rotational velocity data did not contain as much information (e.g., no pre-impact samples) as linear acceleration data and, thus, might act as a source of uncertainty rather than a helpful feature to improve classification performance. However, although our LSTM neural network performed well in identifying VHs when only considering linear acceleration data, future studies should aim towards the collection of accurate rotational velocity data from soccer headers to unveil their additional potential for distinguishing between actual headers and non-header events. Moreover, apart from only using kinematic information, these future approaches may also consider the inclusion of other types of information, such as impact location, in order to potentially improve classification performance.

## Conclusion

In summary, we showed the general ability of a data-driven deep learning network to automatically detect and classify soccer headers based on their characteristic linear acceleration profiles. However, future research should focus on the establishment of techniques to reduce the number of spurious sensor recordings to increase classification precision. This may enable a reliable and time-saving analysis of soccer-related head impacts from wearable sensor data without relying on additional and tedious cross-verification methods, such as video analysis. Within this context, our proposed LSTM classifier may constitute a promising step towards an automatized and exact quantification of individual heading exposure in large-scale studies in order to support both an effective and efficient investigation of the potentially adverse cumulative effects of repetitive soccer heading.

## Supplementary Information

Supplementary Information.

## Data Availability

The datasets generated during and/or analyzed within the current study are available from the corresponding author upon reasonable request.
