# Supplementary material for: A neural network for the detection of soccer headers from wearable sensor data
Source: Sci Rep. 2022 Oct 28;12:18128. doi: 10.1038/s41598-022-22996-2 (PMC9616946; doi:10.1038/s41598-022-22996-2)
Supplement: Supplementary file 1 — Supplementary Information. [file 41598_2022_22996_MOESM1_ESM.pdf]

A Neural Network for the Detection of Soccer Headers from Wearable Sensor Data

Jan Kern*, Thomas Lober, Joachim Hermsdörfer, & Satoshi Endo

Supplementary Information

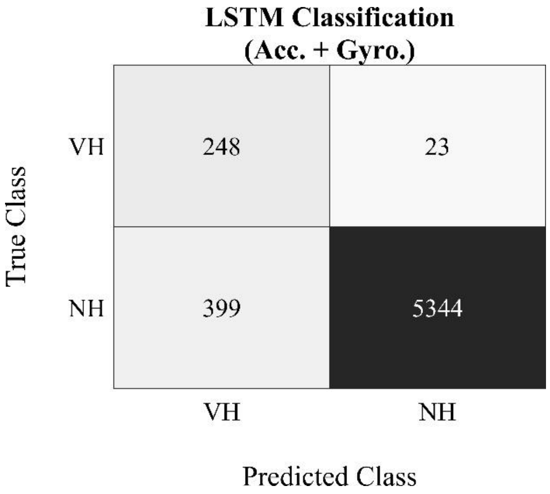

Supplementary Figure S1: Confusion matrix for the LSTM classification of sensor data into soccer headers (VH) and non-headers (NH) based on both linear acceleration and rotational velocity features. Acc: Accelerometer; Gyro: Gyroscope.

Supplementary Table S1: Evaluation of the LSTM neural network's classification performance on an unbalanced dataset (271 VHs, 5743 NHs) based on both linear acceleration and rotational velocity features.

	**LSTM (Acc. + Gyro.)**
**Sensitivity**	91.5 %
**Specificity**	93.0 %
**Precision**	38.3 %
**Accuracy**	93.0 %
**F1**	54.3 %

Acc: Accelerometer; Gyro: Gyroscope.
